# Youth motor competence across stages of maturity: Perceptions of physical education teachers and strength and conditioning coaches

**DOI:** 10.1371/journal.pone.0277040

**Published:** 2022-11-03

**Authors:** Alan M. Burton, Joey C. Eisenmann, Ian Cowburn, Rhodri S. Lloyd, Kevin Till

**Affiliations:** 1 Carnegie School of Sport, Leeds Beckett University, Leeds, United Kingdom; 2 Queen Ethelburga’s Collegiate, York, United Kingdom; 3 Leeds Rhinos Rugby League Club, Leeds, United Kingdom; 4 Youth Physical Development Centre, Cardiff School of Sport, Cardiff Metropolitan University, Cardiff, United Kingdom; 5 Sports Performance Research Institute New Zealand (SPRINZ), AUT University, Auckland, New Zealand; 6 Centre for Sport Science and Human Performance, Waikato Institute of Technology, Hamilton, New Zealand; Mugla Sitki Kocman University: Mugla Sitki Kocman Universitesi, TURKEY

## Abstract

Physical education (PE) teachers and strength and conditioning (S&C) coaches are well placed to develop motor competence within youth populations. However, both groups’ perceptions of important motor competencies are relatively unknown, especially when considering stage of maturity. Therefore, this study aimed to 1) present PE teachers and S&C coaches’ perceptions of motor competence importance according to stage of maturity; 2) compare perceptions of motor competence between stages of maturity, and between PE teachers and S&C coaches; and 3) explore factors that influence PE teachers and S&C coaches’ perceptions of motor competence importance. Via a mixed-method questionnaire, 47 PE teachers (professional experience = 10.3±6.6 years) and 48 S&C coaches (professional experience = 8.6±4.8 years) rated the importance of developing 21 motor competencies across four stages of maturity (childhood, pre-peak height velocity [PHV], circa-PHV, and post-PHV) using a Likert-scale (1 = not important, 5 = very important). Participants also provided open-ended explanations for their perceptions. Frequency analysis indicated that participants rated a broad range of competencies important, with S&C coaches rating more competencies important than PE teachers across all stages of maturity. Mixed-model analysis highlighted several differences in motor competence importance when comparing perceptions between participant groups, and between stages of maturity for PE teachers and S&C coaches. For example, S&C coaches rated strength-based motor competencies less important during childhood (*d* = -1.83 to -0.43), while PE teachers rated them less important during childhood (*d* = -2.22 to -0.42) and pre-PHV (*d* = -1.70 to -0.51) compared to other stages of maturity. Codebook thematic analysis showed several factors that influenced participant’s perceptions of motor competence importance (e.g., participants understanding of themselves). The findings suggest that multiple environments may be required to adequately facilitate motor competence development amongst youth. Coach education should target misunderstandings around the risks of strength-based activity during early stages of maturity and the benefits of developing strength-based motor competencies across youth populations.

## Introduction

Long-term athletic development promotes the holistic development of athleticism (i.e., motor competence, physical fitness and psycho-social characteristics) to improve health, fitness, performance, confidence, and reduce the injury risk of all youth [1]. The term ‘youth’ represents children prior to the onset of puberty (up to ~11 years for girls, and ~13 years for boys) and adolescents following the onset of puberty (typically 12–18 years for girls, and 14–18 years for boys) [1–3]. As an integral component of athleticism, motor competence refers to an individual’s ability to perform a wide range of tasks, where outcomes are underpinned by movement quality, control, and coordination [4–6]. Motor competence consists of multi-dimensional movement capacities (i.e., simple, combined, and complex) that are all inter-related [7, pp. 177–178], and are often categorised into locomotor (e.g., sprinting), object control (e.g., catching), and stability (e.g., balance) skills [8].

Previously, Stodden et al. [9] hypothesised that motor competence interacts with physical fitness and perceived competence (defined by as one’s identification and interpretation of his/her actual motor competence by De Meester et al. [6] and Estevan and Barnett [10]) and influences physical activity levels and weight status across childhood. Others have supported this hypothesis, identifying positive associations between motor competence and physical activity levels [11–13], musculoskeletal strength/endurance [4], cardiorespiratory fitness [4, 11], and inverse associations with weight status [4, 11]. Accordingly, individuals with poor motor competence during childhood may experience reduced actual and perceived motor competence, physical fitness, and physical activity engagement into adolescence and adulthood [14, 15]. Indeed, this could explain poor health and fitness secular trends of youth [16], including increased dropout from organised sports [17, 18], a reduction in youth meeting physical activity guidelines [19] and increasing levels of obesity [20]. Furthermore, this may also contribute to low levels of physical activity in adulthood and subsequent hypokinetic diseases. Therefore, planned programmes which facilitate practice, feedback and instruction [21, 22] are vital for the development and maintenance of motor competence throughout childhood and adolescence [1, 2, 4, 23–25] to foster lifelong participation in physical activity.

To facilitate the development of motor competence, multiple individuals (e.g., physical education [PE] teachers, strength and conditioning [S&C] coaches) should be involved in the planning and delivery of such programmes [2, 26]. Furthermore, recommendations suggest that programmes should be delivered by qualified professionals experienced in training methods, growth and maturation, and pedagogy [1]. Thus, it is imperative to understand the perspectives of such individuals when developing motor competence to help inform coach development, future research and optimise motor competence practices within youth. To date, only S&C coaches’ perspectives of developing motor competence have been evaluated [27]. Burton et al. [27] reported that S&C coaches perceived 69 out of 90 competencies as important (deceleration was most important) and frequently targeted 48 out of 90 competencies (bilateral hip hinge was most practiced). Additionally, analysis showed that S&C coaches who facilitated 3–4 weekly sessions could target 15% and 18% more competencies respectively, compared to coaches who delivered 1–2 sessions per week. Despite these findings, there is a need to broaden our understanding and consider the perspectives of teachers, coaches and other individuals involved in long-term athletic development [1, 2, 26, 27]. Specifically, investigating PE teacher’s perceptions may be insightful as developing motor competence is one of four key aims of the PE curriculum in the United Kingdom, which is compulsory for all children and adolescents until they leave secondary education [28, 29]. From a motor competence perspective, during key stages one and two (i.e., primary education), pupils should be taught basic motor skills (e.g., running, jumping, throwing), how to use these skills in isolation and in combination, and should also develop flexibility, strength, technique, control, and balance through physical activities such as gymnastics [29]. Once in secondary education (i.e., key stages three and four) the focus shifts towards teaching pupils’ techniques through playing competitive sports and focusing on performance during PE lessons [28]. Thus, there appears to be alignment between the PE curriculum and long-term athletic development models (e.g., youth physical development model [30]), which recommend a similar approach of progressing motor competence development from fundamental movement skills towards sports specific skills as youth physically and psychosocially mature. However, S&C coaches do not have a set curriculum, only recommendations from long-term athletic development models, meaning they have more freedom and subjectiveness with what they deliver within their youth programmes. Therefore, understanding and comparing perceptions between coaches and teachers may highlight the need for coach/teacher education and could facilitate collaboration and curriculum reflection to enhance motor competence development for all youths. Additionally, results from Burton et al. [27] failed to account for how S&C coaches’ perceptions may differ according to stage of maturity, which has been highlighted as a critical feature of long-term athletic development [2, 26, 30]. Therefore, it is necessary to explore how perceptions of coaches and teachers differ between stages of maturity (i.e., childhood, adolescent pre-peak height velocity [PHV], adolescent circa-PHV, adolescent post-PHV).

A further limitation of Burton et al. [27] was the lack of qualitative analysis exploring S&C coaches’ perceptions and practices, meaning that findings were relatively descriptive. It is important to understand how and why individuals responsible for facilitating long-term athletic development programmes form their perceptions of motor competence as this could help pinpoint areas to target in future teacher/coach education programmes. Amongst authors, there is a consensus that decision-making is an important component of the coaching process [31–33]. Previous frameworks have highlighted factors that form the decision-making process [31, 32], which have been adapted and applied to S&C [33], and may be applicable to wider coaching environments. As such, the decision-making framework in S&C may form a basis to interpret and understand the perceptions of PE teachers and S&C coaches in more depth, as forming perceptions may follow similar decision-making processes. Therefore, the aims of this study were three-fold: 1) to present PE teachers and S&C coaches’ perceptions of motor competence importance according to stage of maturity; 2) compare perceptions of motor competence between stages of maturity and between individuals (i.e., PE teachers vs. S&C coaches); and 3) explore factors that influence PE teachers and S&C coaches’ perceptions of motor competence importance.

## Materials and methods

### Study design

A concurrent nested quantitative-qualitative mixed-methods questionnaire was adopted for the aims of this study. This approach allowed for a focus on quantitative data (i.e., importance), with a supplementary exploratory element (i.e., why?), and therefore mitigates the lack of qualitative explanations for participant perceptions reported previously [27]. Within the questionnaire, participants reported their perceptions of motor competence across the stages of maturity. The stages of maturity were described in the questionnaire based on the thresholds stated in the Youth Physical Development model for males and females [30] because the model presents stages of maturity for males and females, along with chronological ages ranges where each stage typically occurs. This factor therefore enabled participants who may be unfamiliar with the maturity status concept (e.g., PE teachers as their curriculum aligns towards chronological rather than biological age) to provide perceptions for each stage of maturity. The stages of maturity presented to participants were as follows: Childhood (approximately between 5–11 years for boys, and 5–9 years for girls); adolescent pre-PHV (approximately between 12–14 years for boys, and 10–12 years for girls); adolescent circa-PHV (approximately between 12–16 years for boys, and 10–14 years for girls); and adolescent post-PHV (approximately 16+ years for boys, and 14+ for girls). The questionnaire was developed and administered using Qualtrics^™^ software (Qualtrics, Provo, USA), to PE teachers and S&C coaches working with youths from elite (e.g., talent development) and non-elite (e.g., schools, recreational sports clubs) environments.

### Participants

For this study, UK based PE teachers and S&C coaches were recruited, to allow for comparisons between individuals. Potential participants were given access to the questionnaire through publicly available email addresses, professional networks (e.g., LinkedIn) and social media platforms (e.g., Twitter) as per previous studies [27, 34]. Physical education teachers were required to: 1) be UK-based; 2) hold a relevant teacher training qualification (e.g., post graduate certificate in education); and 3) have a minimum of three years teaching physical education (i.e., ensuring that teachers were of national qualified teacher status or similar, and had a greater history of experience from which to complete the questionnaire). Strength and conditioning coaches were required to: 1) be UK-based, 2) have a minimum of three years’ experience in youth S&C (e.g., coaching children and/or adolescents ≤18 years old); and 3) have accreditation from a relevant governing body (e.g., United Kingdom Strength and Conditioning Association, National Strength and Conditioning Association), and/or a relevant post-graduate qualification (e.g., MSc in Strength and Conditioning). In total, 47 PE teachers (males n = 32, females n = 15; professional experience = 10.3±6.6 years) and 48 S&C coaches (males n = 46, females n = 2; experience = 8.6±4.8 years) were recruited and completed the questionnaire. Participants reported the affiliation which best represented their primary coaching environment. For PE teachers, affiliations reflected the types of schools within the UK, consisting of state primary (n = 6), state secondary (n = 13), academy primary (n = 1), academy secondary (n = 7), independent primary (n = 2), and independent secondary (n = 18) schools. In the UK, state (e.g., community) schools are funded by local authorities and follow the UK’s national curriculum; Academy schools receive funding from local authorities but are run by not-for-profit academy trusts and have more freedom on how they operate than state schools (e.g., they do not have to follow the national curriculum); Independent (i.e., private) schools charge fees to attend instead of receiving government funding, and do not have to follow the national curriculum [35]. Strength and conditioning coaches’ affiliations included team sports (n = 30), individual sports (n = 6), school/multiple sports (n = 10), and higher education (n = 2). This study was granted institutional ethical approval by a University sub-ethics committee (Ref: 81111), with participants providing written consent.

### Identifying and defining motor competencies

The defined motor competencies used for the purpose of this study were adapted from the 90 motor competencies identified by Burton et al. [27]. The 90 motor competencies were condensed down to 21 motor competencies by the lead (AB) and senior researcher (KT). The process involved the lead researcher grouping motor competencies that were considered similar (e.g., acceleration and deceleration = change of speed), into a shortened list of 21 motor competencies. Each of the 21 motor competencies (Table 1) was then defined by the lead researcher and subsequently agreed and confirmed following discussion with the senior researcher.

**Table 1:** List of motor competencies and definitions provided to PE teachers and S&C coaches within the questionnaires.

Motor competency	Definition
Running	Locomotive movements that are faster than walking but slower than sprinting, where both feet are not in contact with the ground at the same time (e.g., Linear running, backwards running, lateral running, running mechanics, curved running).
Sprinting	Running at high/full speed.
Change of speed	A rapid increase or decrease in moving speed (e.g., acceleration, deceleration, or reacceleration).
Change of direction	A movement where the body changes path (e.g., cutting or turning).
Reactive agility	A response to a stimulus that elicits rapid movement of the whole body with change in velocity and/or direction (e.g., dodging).
Jumping	Using both legs to propel the body’s centre of mass in a desired direction after a proceeding counter movement (e.g., horizontal jump, vertical jump, lateral jump, repeated, rebound jump).
Landing	Landing from a jump or fall (e.g., unilateral or bilateral landing).
Hopping and bounding	Repeated jumping on the same (or alternative) leg in a stationary position or to propel the body in a desired direction (e.g., horizontally, laterally).
Core bracing	Engaging the trunk musculature to stabilise the spine and pelvis eliciting or preventing trunk movement (e.g., anti-rotation, flexion, extension, anti-extension, rotation, isometric brace).
Lower body bilateral	Engaging the musculature of the lower body to produce a desired movement (e.g., squat, hinge, hip extension).
Lower body unilateral	Engaging the musculature of 1 leg to perform the desired movement (e.g., unilateral squat, split squat, lunge, unilateral hip hinge, step up).
Upper body pushing	Using the upper body musculature to push the body or an object vertically or horizontally, using 1 or both arms (e.g., press up, shoulder press).
Upper body pulling	Using the upper body musculature to pull the body or an object vertically or horizontally, using 1 or both arms (e.g., pull up, chins).
Mobility	The ability of a joint or body segment to move freely and without restriction (e.g., ankle, hip, thoracic, shoulder).
Advanced weightlifting	Advanced techniques to develop a desired physical adaptation (e.g., Olympic weightlifting, Olympic weightlifting derivatives).
Balance	Stabilising the body’s centre of mass while moving (e.g., walking on a line) or stationary (e.g., standing on 1 leg).
Foundational movements for life	Movements which may be useful throughout life (e.g., swimming, cycling, skating, skiing, crawling, climbing).
Gymnastics and other locomotion	Coordination and stabilisation of the body’s segments while moving (e.g., forward roll, sideways roll, cartwheel, handstand, skipping, vaulting).
Object control	Movements that require the body to control an object using the feet, hands or an implement (e.g., throwing, catching, grasping, kicking, dribbling, striking).
Carrying	Using 1 or both hands to hold an object, or objects, while moving.
Tackling and wrestling	Tackling = Intercepting an object from an opponent or attempting to halt forward progress of an opponent. Wrestling = grappling an opponent while trying to throw or hold them down to the ground.

### Procedures

The questionnaire was presented to PE teachers and S&C coaches in three sections. The first section of the questionnaire collected demographic information relating to years of experience and affiliation (e.g., PE teachers: academy secondary school; S&C coaches: team sport). In section two, participants were presented with the 21 defined motor competencies and were required to rate the importance of developing each motor competency on a 1–5 Likert scale (1 = “not important”; 5 = “very important”) [36] across the four stages of maturity (i.e., childhood, pre-PHV, circa-PHV, post-PHV; [30]). To mitigate the limitations of previous research (e.g., [27]), section three consisted of an open-ended question, which asked participants to explain why they perceived motor competencies as they were reported in section two. This approach was utilised to capture exploratory detail from participants and enhance the study’s methodological rigor. Participants had a maximum of 6-weeks to complete the questionnaire.

### Data analysis

Likert-Scale responses are reported as mean score, standard deviation, and percentages of total responses. For initial analysis, Likert-scale responses were categorised as either “important” (“important” + “very important”) or “not important” (“not important + “little importance”). Qualitative terms were then applied to represent the magnitude of the percentage of total responses as follows: minority = <30%; approximately a third = ~ 30%; approximately half = ~50%; majority = 55–74%; most = ≥75%; all = 100% of PE teachers/S&C coaches, in line with previous research [27, 37, 38].

To evaluate the differences between PE teachers and S&C coaches perceived importance, and by stage of maturity, Likert-scale responses were log-transformed to a continuous scale (i.e., converted from 1–5 scale to 0–100% scale), analysed using linear mixed models, and back transformed to present the results. A linear mixed model was conducted for each motor competency, with job title (PE teacher or S&C coach) and stage of maturity (childhood, pre-PHV, circa-PHV, post-PHV) as fixed factors. For each mixed model, the interaction between job title and stage of maturity was compared firstly by stage of maturity (for PE teacher and S&C responses separately) and then compared by job title (i.e., PE teachers vs. S&C coaches), using a Bonferroni post-hoc adjustment. Effect sizes were used to show the magnitude of difference in perceptions between participants and stage of maturity, which was reported as *d* ± 95% confidence intervals. Thresholds for effect sizes were set as follows: 0–0.19, trivial; 0.2–0.59, small; 0.6–1.19, moderate; > 1.2, large [39].

Thematic analysis, which included template deductive and inductive elements, was conducted by the lead researcher to evaluate qualitative responses from participants. Physical education teacher’s and S&C coaches’ responses were analysed together as the exploratory question aimed to understand the similarities and differences in the quantitative responses given by participants. The template deductive element utilised the decision-making framework in S&C [33] as a template for the first order themes [40]. The lead researcher familiarised themselves with the responses, whilst checking for any spelling or typographical errors. Elements within the text that could be encompassed within one of the initial themes were marked and noted [40]. Following this, an inductive stage involved identifying interesting features within the data and grouping similarly related features [41]. These collated codes were then categorised into a general second order theme (e.g., explanations referencing sport(s) were categorised into a “sport” second order theme). Second order themes deemed to include further detail were sub-grouped into specific third order themes (e.g., within the “sport” second order theme, explanations were grouped into “influenced by sports participation”, or “bias towards sports coached”). The second and third order themes were finally reviewed against the coding template to confirm which first-order theme they represented.

## Results

### PE teacher’s and S&C coaches’ perceived importance of motor competencies

The means, standard deviations, and percentage of total responses for perceived importance of motor competencies for PE teachers and S&C coaches are presented in Table 2. Most (≥ 75%) Physical Education teachers rated four competencies important (reported a score of four or five) at childhood, four at pre-PHV, three at circa-PHV and two at post-PHV. A further six, nine, 11, and 13 motor competencies were rated important by the majority (55–74%) of PE teachers at childhood, pre-PHV, circa-PHV, and post-PHV, respectively. Seven motor competencies were rated important by the majority of PE teachers across all stages of maturity. Most S&C coaches deemed seven competencies important at childhood, 11 at pre-PHV, 15 at circa-PHV, and 14 at post-PHV as important. During childhood, pre-PHV, circa-PHV and post-PHV, the majority of S&C coaches perceived a further five, seven, two and three motor competencies important, respectively. Neither PE teachers nor S&C coaches perceived resistance training movements (e.g., lower body bilateral, upper body pushing/pulling) important in childhood. Tackling and wrestling were not rated important by PE teachers at any stage of development (<55% scored 4 or 5), whilst carrying was not classified as important by S&C coaches. S&C coaches rated a total of nine motor competencies as important across all stages of maturity.

**Table 2:** PE teachers and S&C coaches’ percentage of response frequencies for motor competencies at different stages of maturity.

	Childhood				Pre-PHV				Circa-PHV				Post-PHV			
	PE teachers		S&C coaches		PE teachers		S&C coaches		PE teachers		S&C coaches		PE teachers		S&C coaches	
Movement	Mean ± SD	% 4 or 5	Mean ± SD	% 4 or 5	Mean ± SD	% 4 or 5	Mean ± SD	% 4 or 5	Mean ± SD	% 4 or 5	Mean ± SD	% 4 or 5	Mean ± SD	% 4 or 5	Mean ± SD	% 4 or 5
Running	4.7 ± 0.7	85**	4.4 ± 0.9	88^^	4.4 ± 0.7	70*	4.4 ± 0.7	85^^	4.2 ± 0.8	61*	4.5 ± 0.6	94^^	4.0 ± 1.1	55*	4.1 ± 1.2	79^^
Sprinting	3.4 ± 1.2	31	4.0 ± 1.1	73^	4.0 ± 0.9	49	4.4 ± 0.8	83^^	4.4 ± 0.7	70*	4.6 ± 0.6	92^^	4.3 ± 0.9	68*	4.8 ± 0.6	98^^
Change of speed	3.7 ± 1.0	40	3.9 ± 1.1	63^	4.2 ± 0.7	57*	4.2 ± 0.9	81^^	4.3 ± 0.7	66*	4.5 ± 0.8	90^^	4.4 ± 0.9	73*	4.7 ± 0.8	94^^
Change of direction	4.1 ± 1.1	60*	4.0 ± 1.0	71^	4.4 ± 0.7	69*	4.3 ± 0.8	81^^	4.5 ± 0.6	76**	4.6 ± 0.6	96^^	4.3 ± 0.9	66*	4.7 ± 0.6	98^^
Reactive agility	3.9 ± 1.2	53	3.9 ± 1.1	65^	4.2 ± 0.9	62*	4.1 ± 0.9	73^	4.2 ± 0.7	61*	4.4 ± 0.8	79^^	4.1 ± 0.9	55*	4.6 ± 0.9	92^^
Jumping	4.4 ± 0.8	71*	4.4 ± 0.8	83^^	4. 5 ±0.7	75**	4.4 ± 0.6	92^^	4.2 ± 0.8	59*	4.5 ± 0.8	88^^	4.0 ± 0.9	48	4.6 ± 0.8	94^^
Landing	4.3 ± 0.9	66*	4.6 ± 0.6	92^^	4.3 ± 0.7	65*	4.6 ± 0.6	94^^	4.3 ± 0.7	64*	4.6 ± 0.5	98^^	4.1 ± 0.9	56*	4.3 ± 1.0	85^^
Hopping & bounding	4.0 ± 1.0	53	3.8 ± 1.0	60^	4.3 ± 0.7	62*	4.1 ± 0.9	69^	4.1 ± 0.8	55*	4.2 ± 0.8	83^^	4.0 ± 0.9	53	4.4 ± 0.9	92^^
Core bracing	3.4 ± 1.2	36	3.4 ± 1.0	42	3.8 ± 1.0	45	3.8 ± 0.8	65^	4.2 ± 0.9	61*	4.3 ± 0.7	85^^	4.2 ± 0.9	62*	4.3 ± 1.0	85^^
Lower body bilateral	3.0 ± 1.2	21	3.7 ± 1.0	54	3.8 ± 0.9	42	4.2 ± 0.8	77^^	4.4 ± 0.6	67*	4.4 ± 0.8	85^^	4.5 ± 0.8	75**	4.5 ± 0.9	92^^
Lower body unilateral	2.9 ± 1.1	17	3.7 ± 1.0	54	3.6 ± 0.9	34	4.2 ± 0.8	77^^	4.0 ± 0.9	53	4.6 ± 0.7	90^^	4.3 ± 0.9	67*	4.6 ± 0.8	94^^
Upper body pushing	2.5 ± 1.1	10	3.4 ± 1.1	44	3.3 ± 1.0	26	3.8 ± 0.9	56^	4.1 ± 0.8	54	4.3 ± 0.7	83^^	4.4 ± 0.8	73*	4.3 ± 1.0	88^^
Upper body pulling	2.4 ± 1.0	8	3.4 ± 1.1	48	3.3 ± 1.1	25	3.9 ± 0.9	60^	4.0 ± 0.9	51	4.3 ± 0.7	88^^	4.3 ± 1.0	70*	4.4 ± 0.9	90^^
Mobility	3.8 ± 1.4	57*	3.1 ± 1.2	33	4.1 ± 1.0	59*	3.8 ± 1.0	60^	4.4 ± 0.7	67*	4.4 ± 0.8	85^^	4.5 ± 0.8	75**	4.4 ± 0.9	92^^
Advanced weightlifting	1.1 ± 0.3	0	1.7 ± 0.9	2	1.6 ± 0.8	0	2.4 ± 1.1	19	2.4 ± 1.1	7	3.2 ± 1.2	42	3.4 ± 1.2	32	3.5 ± 1.3	63^
Balance	4.7 ± 0.7	87**	4.3 ± 1.0	85^^	4.7 ± 0.5	84**	4.1 ± 0.9	75^^	4.5 ± 0.8	76**	4.1 ± 0.9	75^^	4.3 ± 0.9	68*	3.3 ± 1.3	54
Foundation movements for life	4.6 ± 0.7	81**	4.6 ± 0.9	83^^	4.7 ± 0.5	85**	4.2 ± 0.9	75^^	4.5 ± 0.7	76**	3.7 ± 1.0	52	4.3 ± 0.9	64*	2.8 ± 1.3	35
Gymnastics and other locomotion	4.4 ± 0.7	71*	4.6 ± 0.7	90^^	4.2 ± 0.8	62*	4.1 ± 0.9	73^	3.7 ± 0.9	36	3.6 ± 1.0	54	3.4 ± 1.0	28	2.7 ± 1.3	33
Object control	4.5 ± 1.0	78**	4.7 ± 0.7	94^^	4.6 ± 0.7	77**	4.4 ± 0.8	85^^	4.3 ± 0.8	66*	4.1 ± 1.0	71^	4.1 ± 0.9	57*	3.5 ± 1.4	60^
Carrying	4.2 ± 1.1	65*	3.5 ± 1.2	52	4.0 ± 1.0	56*	3.6 ± 1.0	52	3.6 ± 1.2	43	3.4 ± 1.1	46	3.4 ± 1.2	34	3.0 ± 1.4	46
Tackling and wrestling	2.0 ± 1.2	9	2.9 ± 1.2	29	2.7 ± 1.3	19	3.1 ± 1.1	38	3.2 ± 1.2	26	3.5 ± 1.1	56^	3.4 ± 1.2	36	3.5 ± 1.3	67^

* Motor competency rated "important" (scored 4 or 5) by the majority (55–74%) of PE teachers;

** Motor competency rated "important" (scored 4 or 5) by most (≥75%) PE teachers;

^ Motor competency rated "important" (scored 4 or 5) by the majority (55–74%) of S&C coaches;

^^ Motor competency rated "important" (scored 4 or 5) by most (≥75%) S&C coaches. PHV = Peak height velocity.

### Differences in perceived motor competency importance between stages of maturity, and between PE teachers and S&C coaches

Linear mixed models for the interaction between participant groups and stage of maturity highlighted significant findings for the following types of motor competencies: running (F_3,279_ = 3.33, *p*<0.05), change of direction (F_3,279_ = 3.35, *p*<0.05), jumping (F_3,279_ = 6.15, *p*<0.01), hopping and bounding (F_3,279_ = 4.03, *p*<0.01), lower body bilateral (F_3,279_ = 6.46, *p*<0.01), lower body unilateral (F_3,279_ = 2.69, *p*<0.05), upper body pushing (F_3,279_ = 11.24, *p*<0.01), upper body pulling (F_3,279_ = 10.26, *p*<0.01), mobility (F_3,279_ = 5.73, *p*<0.01), advanced weightlifting (F_3,279_ = 5.12, *p*<0.01), balance (F_3,279_ = 4.56, *p*<0.01), foundational movements for life (F_3,279_ = 18.24, *p*<0.01), gymnastics and other locomotion (F_3,279_ = 5.90, *p*<0.01), object control (F_3,279_ = 5.63, <0.01), and tackling and wrestling (F_3,279_ = 4.93, *p*<0.01). Models were statistically insignificant (*p*>0.05) for sprinting, reactive agility, landing, core bracing, and carrying motor competencies.

### Differences between stages of maturity

#### PE teachers

**Fig 1:**
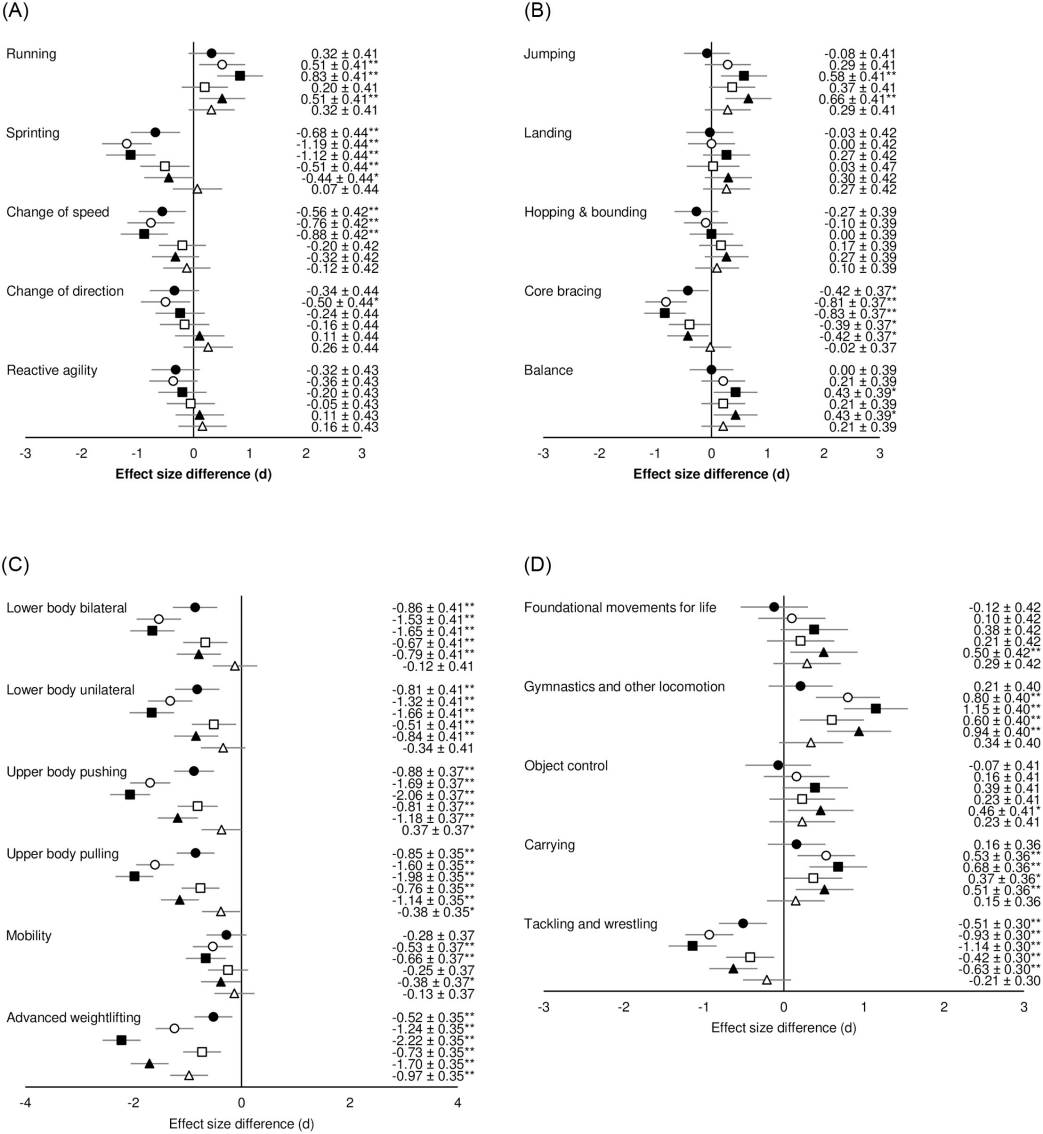
**A.** Effect size differences in PE teacher’s perceived motor competence importance between stages of maturity for running-based competencies. **B.** Effect size differences in PE teacher’s perceived motor competence importance between stages of maturity for jumping and stability competencies. **C.** Effect size differences in PE teacher’s perceived motor competence importance between stages of maturity for strength-based competencies. **D.** Effect size differences in PE teacher’s perceived motor competence importance between stages of maturity for other fundamental/foundational competencies. Black circles = childhood vs. pre-PHV; white circles = childhood vs. circa-PHV; black squares = childhood vs. post-PHV; white squares = pre- vs. circa-PHV; black triangles = pre- vs. post-PHV; white triangles = circa- vs. post-PHV. Negative effect sizes = less important (i.e., if the comparison is “a” vs. “b”, and effect size is negative, then the motor competency is less important during “a” compared to “b”); positive effect size = more important (i.e., if the comparison is “a” vs. “b”, and effect size is positive, then the motor competency is more important during “a” compared to “b”). *p <0.05, ** *p* <0.01.

Post-hoc analysis comparing PE teacher’s perceptions by stage of maturity revealed several differences in the importance of motor competencies. The effect size differences for running-based competencies are presented in Fig 1A. Two competencies were significantly less important during childhood than pre-PHV, three competencies significantly less important during childhood vs. circa-PHV, and two competencies were less important during childhood compared to post-PHV. “Sprinting” was perceived as less important during pre-PHV compared to circa-, and post-PHV. “Running” was perceived as more important during childhood compared to circa-, and post-PHV, and more important during pre-PHV vs. post-PHV. The difference in importance for “Sprinting” presented the larges effect size (*d* = -1.19, *p*<0.01) and was less important during childhood compared to circa-PHV.

For jumping and stability competencies, “jumping” and “balance” were perceived as significantly more important during childhood and pre-PHV compared to post-PHV. “Core bracing” was perceived as less important during childhood compared to pre-, circa-, and post-PHV, as well as pre-PHV compared to circa-, and post-PHV. The largest difference was for “core bracing” which was less important during childhood compared to post-PHV (*d* = -0.83, *p*<0.01). All effect size differences for PE teacher’s perceptions of jumping and stability competencies are presented in Fig 1B.

Differences in PE teacher’s perceptions for strength-based competencies are presented in Fig 1C, with significant differences ranging from small to large. Five out of six strength-based competencies were perceived as significantly less important during childhood compared to pre-PHV, with all competencies perceived as less important during childhood compared to circa-, and post-PHV. Similarly, five strength-based competencies were perceived as less important during pre-PHV compared to circa-PHV, while all competencies were perceived as less important during pre-PHV compared to post-PHV. Four competencies were perceived as less important during circa-PHV compared to post-PHV. “Advanced weightlifting” had the highest difference in importance and was perceived as more important during childhood compared to post-PHV (*d* = -2.22, *p*<0.01).

Significant effect size differences in PE teachers perceived importance of other fundamental/foundational competencies between stages of maturity ranged from small to moderate (Fig 1D). Two competencies were perceived as more important during childhood compared to circa-, and post PHV, with differences being small to moderate. Similarly, two competencies were significantly more important during pre-PHV than circa-PHV. Four out of five competencies were perceived as more important during pre-PHV compared to post-PHV. “Tackling and wrestling” was the only fundamental/foundational competency which was perceived as less important during childhood compared to all other stages of maturity, as well as being perceived as less important during pre-PHV compared to circa- and post-PHV. The greatest difference in importance was between childhood and post-PHV for “gymnastics and other locomotion” (*d* = 1.15, *p*<0.01; more important during childhood).

#### S&C coaches

**Fig 2:**
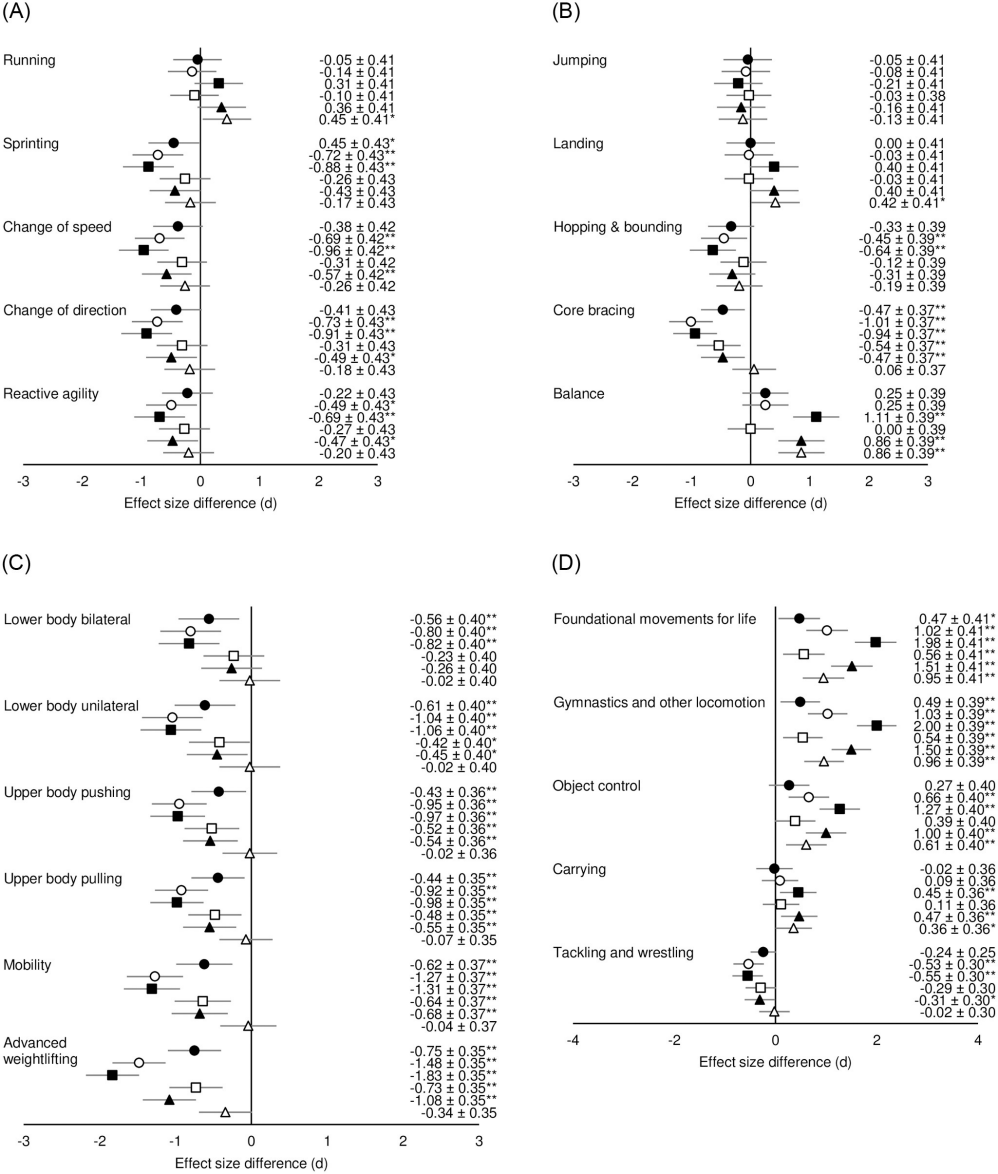
**A.** Effect size differences in PE teacher’s perceived motor competence importance between stages of maturity for running-based competencies. **B.** Effect size differences in PE teacher’s perceived motor competence importance between stages of maturity for jumping and stability competencies. **C.** Effect size differences in PE teacher’s perceived motor competence importance between stages of maturity for strength-based competencies. **D.** Effect size differences in PE teacher’s perceived motor competence importance between stages of maturity for other fundamental/foundational competencies. Black circles = childhood vs. pre-PHV; white circles = childhood vs. circa-PHV; black squares = childhood vs. post-PHV; white squares = pre- vs. circa-PHV; black triangles = pre- vs. post-PHV; white triangles = circa- vs. post-PHV. Negative effect sizes = less important (i.e., if the comparison is “a” vs. “b”, and effect size is negative, then the motor competency is less important during “a” compared to “b”); positive effect size = more important (i.e., if the comparison is “a” vs. “b”, and effect size is positive, then the motor competency is more important during “a” compared to “b”). *p <0.05, ** *p* <0.01.

Post-hoc analysis showed S&C coaches’ perceptions of motor competency importance differed between stages of maturity. A Small difference showed that S&C coaches perceived “running” more important during circa-PHV compared to post-PHV. “Sprinting” was perceived as less important during childhood compared to all other stages of maturity, while three other running-based competencies were less important during childhood vs. circa-, and post-PHV (small to moderate differences). The same three competencies were also less important during pre-PHV compared to post-PHV (small differences). The greatest difference in importance was “change of speed” which was perceived as less important during childhood compared to post-PHV (*d* = -0.96, *p*<0.01; Fig 2A).

For jumping and balance competencies (Fig 2B), two competencies were more important during childhood competed to post-PHV, with differences being small to moderate. “Balance” was more important during pre-PHV compared to circa-, and during circa-PHV vs. post-PHV (moderate differences). “Landing” was also perceived as more important during circa-PHV and post-PHV (small difference). During childhood, “core bracing” was less important than pre-, circa, and post PHV, and was also less important during pre-PHV vs. circa-, and post-PHV (small to moderate differences). “Hopping & bounding” was also less important during childhood compared to circa- and post-PHV (small and moderate differences respectively). “Balance” had the greatest difference in importance and was less important during childhood compared to post-PHV (*d* = -1.11, *p*<0.01).

Small to large differences showed that all strength-based motor competencies were perceived as less important during childhood compared to pre-, circa-, and post-PHV. Five out of six strength-based competencies were also perceived as less important during pre-PHV vs. circa-, and post-PHV (small to moderate differences). “Advanced weightlifting” presented the greatest difference in importance and was less important during childhood compared to post-PHV (*d* = -1.83, *p*<0.01; Fig 2C).

For other fundamental/foundational competencies (Fig 2D), two competencies were perceived as more important during childhood compared to all other stages of maturity, more important during pre-PHV than during circa- or post-PHV, and more important during circa-PHV compared to post-PHV (small to large differences). “Object control” was perceived more important during childhood compared to circa- or post-PHV, and more important during pre-PHV compared to post PHV (moderate to large differences). Similarly, “carrying” was more important during childhood compared post-PHV, and more important during pre-PHV vs. post-PHV (small differences). “Tackling and wrestling” was perceived as less important during childhood competed to circa-, and post-PHV (small differences). The greatest effect size difference was for “gymnastics and other locomotion” which was more important during childhood compared to post-PHV (*d* = 2.00, *p*<0.01).

### Differences between PE teacher and S&C coach responses at different stages of development

**Fig 3:**
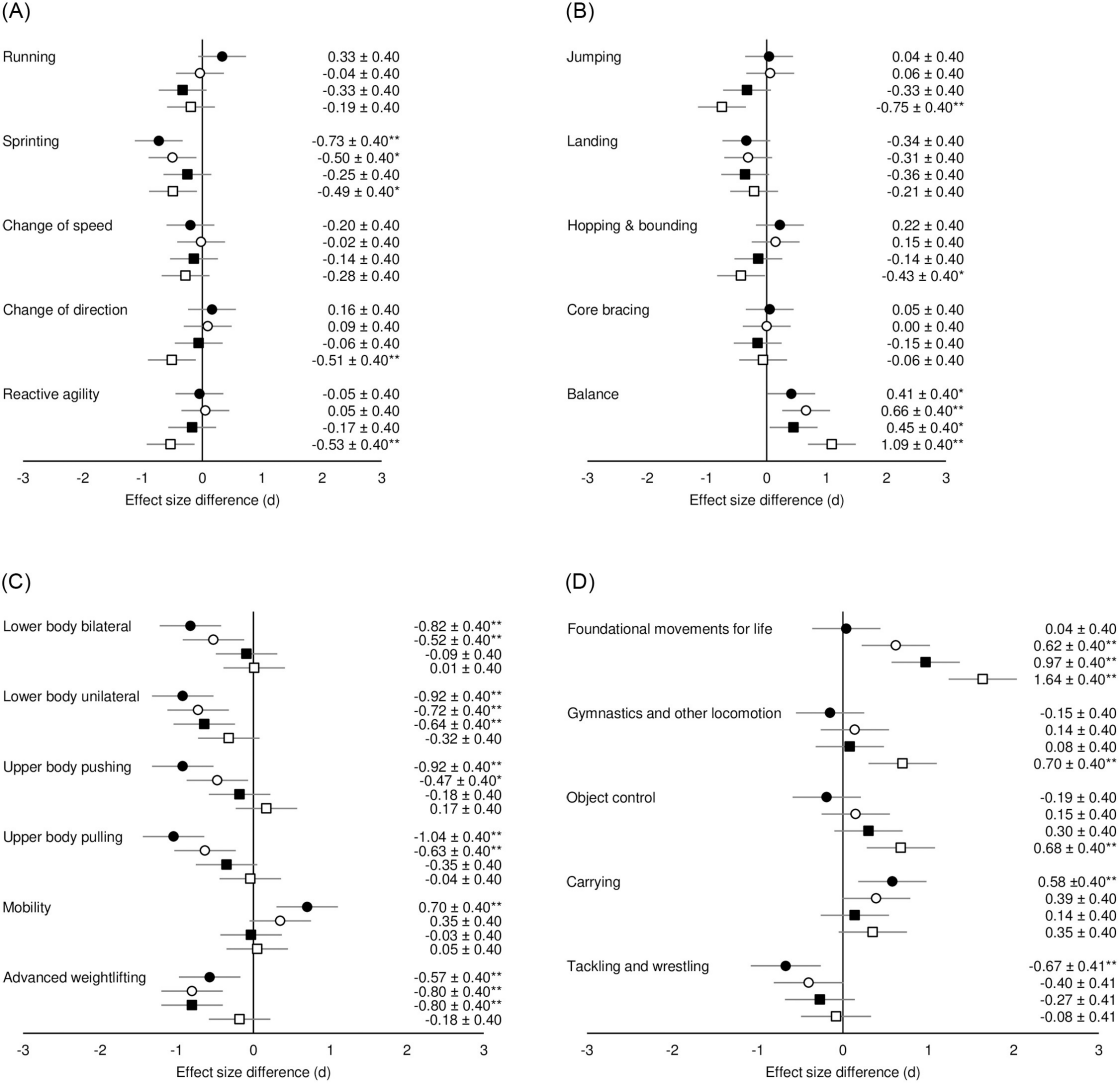
**A.** Effect size differences in perceived motor competence importance between PE teachers and S&C coaches across stages of maturity for running-based competencies. **B.** Effect size differences in perceived motor competence importance between PE teachers and S&C coaches across stages of maturity for jumping and stability competencies. **C.** Effect size differences in perceived motor competence importance between PE teachers and S&C coaches across stages of maturity for strength-based competencies. **D.** Effect size differences in perceived motor competence importance between PE teachers and S&C coaches across stages of maturity for other fundamental/foundational competencies. Black circles = childhood; white circles = pre-PHV; black squares = circa-PHV; white squares = post-PHV. Negative effect size = PE teachers rated the motor competency less important than S&C coaches; positive effect size = PE teachers rated the motor competency more important than S&C coaches. *p <0.05, ** *p* <0.01.

Post-hoc analysis showed several differences between PE teachers and S&C coaches’ responses across stages of maturity. For running-based competencies (Fig 3A), PE teachers rated “sprinting” less important than S&C coaches during childhood and pre-PHV, as well as three competencies during post-PHV (small to moderate differences). The running-based competency with the greatest difference between PE teacher’s and S&C coaches’ perceptions was for “sprinting” during childhood (*d* = -0.73, *p*<0.01; rated less important by PE teachers compared to S&C coaches).

Effect size differences for PE teachers and S&C coaches’ perceptions of Jumping and stability competencies are presented in Fig 3B. Moderate and small differences were present for “jumping” and “hopping & bounding” respectively during post-PHV, with PE teachers rating these competencies less important than S&C coaches. PE teachers rated “balance” significantly more important than S&C coaches across all stages of maturity, which was also presented as the greatest effect size difference during post-PHV (*d* = 1.09, *p*<0.01).

Five out of six strength-based competencies were perceived as less important by PE teachers compared to S&C coaches during childhood and pre-PHV, with differences ranging from small to moderate. Moderate differences also highlighted two competencies which PE teachers rated as less important than S&C coaches during circa-PHV. PE teachers rated “mobility” more important than S&C coaches during childhood. The strength-based competency which presented the greatest effect size difference between PE teachers and S&C coaches was “upper body pulling” during childhood (*d* = -1.04, *p*<0.01; rated less important by PE teachers compared to S&C coaches; Fig 3C).

Fig 3D shows the effect size differences between PE teachers and S&C coaches’ perceptions of other fundamental/foundational competencies. During childhood, PE teachers rated “tackling and wrestling” less important, and “carrying” more important, competed to S&C coaches. “Foundation movements for life” were perceived as more important by PE teachers compared to S&C coaches during pre-, circa-, and post-PHV. PE teachers also rated a further two competencies more important than S&C coaches during post-PHV. “Foundational movements for life” presented the greatest difference between perceptions during post-PHV, where PE teachers rated the competency more important than S&C coaches (*d* = 1.64, *p*<0.01).

### Explanations for motor competence perceptions

The analysis of open-ended responses (PE teachers n = 22; S&C coaches n = 10) resulted in four first-order themes that described the reasons behind participants perceptions: a) PE teachers and S&C coaches understanding of themselves; b) What PE teachers and S&C coaches Coach–Their Curriculum; c) Who PE teachers and S&C coaches Coach–Their Participants; and d) How PE teachers and S&C coaches Coach–Their Pedagogy. First-order themes and accompanying second- and third-order themes, along with representative quotes are highlighted in Table 3.

**Table 3:** Hierarchical themes representing PE teachers and S&C coaches’ reasons for their perceptions of motor competency importance.

First order themes	Second order themes	Third order themes	Representative quote
PE teachers and S&C coaches understanding of themselves	Existing knowledge	Training principles	S&C coach 36: “Due to the diminishing returns principle, greater specificity or more "advanced training" methods may be required to stimulate improvements in athleticism”.
		Textbook/taught knowledge	S&C coach 13: “In terms of which are most important ("5"), there is a shift from the most fundamental movements in childhood to then extend on those building blocks to movements that are a little more challenge and/or a little more directly linked to sports performance/training”.
		Injury prevention/risk reduction	PE teacher 1: “Everything like landing, agility are still important to avoid injury…”.
		Myths/misconceptions	PE teacher 2: “The only ones I would question could be the ones in the younger category that may have encouraged body adaptation before the body has fully developed”.
			PE teacher 24: “Risk of injury or stunted growth”.
	Existing beliefs and values	General beliefs and values	PE teacher 2: “I generally think most of these skills are extremely important in the development of motor skills for all ages”.
			PE teacher 29: “Pure body weight activities in my opinion is all that is required until mid to late teens when this can be accompanied with extra resistance”.
		Importance of foundations/building blocks/fundamentals	PE teacher 1: “Youth need to work on the basic fundamental movements—things like gymnastics, fun games, hopping basic exercises can help form a range of movement…”.
			PE teacher 16: “I think the fundamental movements of running, jumping, balance, mobility are importance across all ages”.
			S&C coach 30: “Fundamental movement skills vital during childhood”.
		The need to progress/develop/transfer	PE teacher 23: “I believe fundamental skills should be taught throughout regardless of age as each has a progression and allows for transfer of skill”.
			S&C coach 22: “Each stage of the LTAD in my own philosophy should develop from previous stages… have a core of movements… once you get into adolescence you can bring in more complexity”.
		The need to reduce injury risk	PE teacher 23: “Skill such as landing should hold great importance to allow for longevity in sport”.
			PE teacher 35: “Many accidents happen because people are not agile enough or cannot hold themselves in a balanced and solid position…”.
	Past experiences	Demographic changes	PE teacher 30: “Had I been filling in this 20–30 years ago I would not have been putting in as important for Adolescent Circa and Post… there has been a very noticeable decline in children’s motor skills, which has resulted in more time being needed to foster these at secondary level… The decline in motor competency over the years I have been teaching is alarming”.
		Observations	PE teacher 7: “The children that have been getting the appropriate age-related instruction in the fundamental move patterns / ABC’s are without doubt more likely to be the more inclined to try different aspects of the subject”.
	Reflective practice		PE teacher 7: “Questioning why we are doing things and how…”.
What PE teachers and S&C coaches coach–their curriculum	Sport/activities coached	General physical activity/sport/physical literacy	PE teacher 35: “Much of the motor skills listed are imperative… so children are aware of how to engage muscles and take part in physical activity safely”.
			S&C coach 36: “Locomotion and the technical skill of movements underpin the athlete’s ability to meet the technical, tactical and movement demands of their sport(s); therefore, these have the highest priority”.
			S&C coach 34: “…the need is to develop the child holistically to prepare for life and global sporting activities in the pursuit of physical literacy”.
		Physical Education	PE teacher 27: “Contact based work is too specific and therefore I would say not needed as part of any PE”.
		Sport specific	S&C coach 23: “bias from rugby”.
			S&C coach 28: “I work with football players and so the movement tasks related to football execution maintain importance throughout”.
Who PE teachers and S&C coaches coach–their participant’s	Prescriptive approach to developing youth	Perceptions influenced by stage of maturity	PE teacher 20: “Specific motor skills also require maturity levels with development and therefore will only be discovered later on in life”.
			S&C coach 13: “…by post-PHV they have developed the skills to undertake high quality S&C training.”.
		Perceptions influenced by chronological age	PE teacher 14: “By 14+ I’d like to think they were well versed in the FMS and we can focus on more advanced movements…”.
			S&C coach 30: “…emphasis gradually moves to sports specific skills as the athlete ages”.
	Responsive approach to developing youth	Perceptions influenced by physical capabilities	S&C coach 36: “These movements are determined by the athlete’s physical capabilities to generate (force -velocity-power); hence, the development of the fundamental skills to be able safely and effectively train these qualities (i.e., resistance training) is of great importance”.
		Perceptions influenced by physical and psycho-social capabilities	PE teacher 24: “whether or not a young person is at the stage to physically and mentally cope with the demands of that motor competency”.
How PE teachers and S&C coaches coach–their pedagogy	Coaches’ methods	Fun games	PE teacher 29: “I feel there is a need to expose younger children to technical aspects of the above in fun and engaging activities to develop a core fundamental base without an importance of result- based outcomes”.
			S&C coach 39: “skills such as COD and Reactive Agility are vital, but RA can be delivered via fun games”.
		General coaching methods	PE teacher 7: “… getting the child to think like an athlete only helps to engage them in the process of teaching and learning”.
			S&C coach 39: “I believe if we can ingrain that (running technique) early and continue to micro dose…”.
			S&C coach 24: “For childhood, just exposing athletes to a wide variety of movement patterns is crucial, not one specific movement. Coaching doesn’t have to be very technical at all”.
			PE teacher 7: “Also teaching PE as a subject, and not just a "PE lesson", also has a massive effect on outcomes, really working towards lesson objectives with a purpose”.
			S&C coach 13: “How the skills are coached… in practice skills would being to be linked to more mature athletes can combine movements together”.

#### PE teachers and S&C coaches understanding of themselves

This theme represents participants’ knowledge, values and beliefs, previous experience, and reflective practice. Physical education teachers and S&C coaches understanding of themselves was the most highlighted explanation for perceptions of motor competency importance by PE teachers and S&C coaches. The reflective practice sub-theme helps to explain this first-order theme. For example, PE teacher 7 stated that “*questioning why we do things and how*…” influenced their perceptions. Whilst identified as a sub-theme, this point highlights that participants’ understanding of themselves is supported by a reflective process that enhances their knowledge, values, and beliefs.

Existing knowledge, as a second-order theme, represents how PE teachers and S&C coaches combine and interpret their knowledge across all aspects of decision-making (e.g., who, what, how, context) and apply this within their practices. Participants highlighted existing knowledge of training principles, textbook theory, and importance of injury risk reduction, as shown by PE teacher 1 “*everything like landing*, *agility are still important to avoid injury*…”. Furthermore, a myth/misconception theme was identified based upon quotes from PE teacher 2 “*The only ones I would question could be the ones in the younger category that may have encourage body adaptation before the body has fully developed*.”, and PE teacher 24 “*Risk of injury or stunted growth*.”. These quotes were deemed myths/misconceptions rather than outdated knowledge because of similarities with the myths associated with resistance training highlighted by Faigenbaum and McFarland [42], which are disproved by evidence which suggests that the risks associated with resistance training are no greater than everyday activities [43, 44]. Such myths align with the lack of strength-based motor competencies (e.g., lower body bilateral/unilateral) rated as important during childhood and pre-PHV.

The existing values and beliefs sub-theme is represented by quotes where participants discuss their own beliefs, values, and philosophies. Both PE teachers and S&C coaches believed that they should develop a broad range of motor competencies during childhood, and progress towards more advanced competencies as suggested by S&C coach 22 *“each stage of the LTAD in my own philosophy should develop from previous stages*… *have a core of movements*… *once you get into adolescence you can bring in more complexity”*.

Past experiences represents where participants provided anecdotal detail of their previous experiences and was commented on by two PE teachers. One teacher suggested that demographic changes have altered their perceptions to favour the importance of motor competencies during adolescence,

“*Had I been filling in this 20–30 years ago I would not have been putting in as important for Adolescent Circa and Post*… *there has been a very noticeable decline in children’s motor skills*, *which has resulted in more time being needed to foster these at secondary level*… *The decline in motor competency over the years I have been teaching is alarming*”

(PE teacher 30).

A second teacher (PE teacher 7) indicated a general observation whereby children who develop key motor competencies appear to participate in wider aspects of PE, *“The children that have been getting the appropriate age-related instruction in the fundamental move patterns / ABC’s are without doubt more likely to be the more inclined to try different aspects of the subject”*, suggesting a greater importance towards developing motor competencies during childhood.

What PE teachers and S&C coaches coach–their curriculum. This theme represents responses where PE teachers and S&C coaches indicated that their perceptions are influenced by the activities they coach. For example, responses were grouped into quotes referring to general activity and sport *“Much of the motor skills listed are imperative… so children are aware of how to engage muscles and take part in physical activity safely”* (PE teacher 35), *“Locomotion and the technical skill of movements underpin the athlete’s ability to meet the technical*, *tactical and movement demands of their sport(s); therefore*, *these have the highest priority”* (S&C coach 36), PE *“Contact based work is too specific and therefore I would say not needed as part of any PE”* (PE teacher 27), or sport specific activities *“I work with football players and so the movement tasks related to football execution maintain importance throughout”* (S&C coach 28).

#### Who PE teachers and S&C coaches coach–their participants

The theme “who PE teachers and S&C coaches coach–their participants” represents responses where participants take a prescriptive or responsive approach to developing youth. The prescriptive approach represents PE teachers and S&C coaches’ knowledge, experience and expectations of what competencies should be developed at a particular stage of maturity, *“…by post-PHV they have developed the skills to undertake high quality S&C training”* (S&C coach 13), or biological age, *“by 14+ I’d like to think they were well versed in the FMS and we can focus on more advanced movements…”* (PE teacher 14). The responsive approach refer to what participants perceive important based on their individuals capabilities, for example physical capabilities *“These movements are determined by the athlete’s physical capabilities to generate (force -velocity-power); hence*, *the development of the fundamental skills to be able safely and effectively train these qualities (i*.*e*., *resistance training) is of great importance”* (S&C coach 36) or physical and psycho-social capabilities *“whether or not a young person is at the stage to physically and mentally cope with the demands of that motor competency”* (PE teacher 24).

How PE teachers and S&C coaches coach–their pedagogy. This theme represents responses from PE teachers and S&C coaches that discuss their approaches to developing motor competence when explaining their perceptions. Fewer participants highlighted pedagogical approaches within explanations for their perceptions, although various pedagogical approaches for developing motor competence were highlighted (e.g., fun games, micro-dosing, the inclusion of psychosocial elements, combining skills). Both PE teachers and S&C coaches referenced the use of fun games as pedagogical approaches, and suggest they are favoured during early stages of maturity (e.g., PE teacher 29: “*I feel there is a need to expose younger children to technical aspects of the above in fun and engaging activities*…”). As such this may indicate why perceptions differ between stages of maturity.

## Discussion

The development of motor competence within youth is vital for health and performance benefits [1]. To improve motor competence within youth, it is key to understand the perspectives of multiple individuals responsible for long-term athletic development, compare perceptions, and evaluate these perceptions against existing recommendations. Therefore, the aims of this study were to: 1) present PE teachers and S&C coaches’ perceptions of motor competence importance according to stage of maturity (i.e., childhood, pre-PHV, circa-PHV, post-PHV); 2) compare PE teachers and S&C coaches’ perceptions of motor competence between stages of maturity, and compere perceptions between PE teachers and S&C coaches at each stage of maturity; and 3) explore factors that influence PE teachers and S&C coaches perceptions of motor competence importance. The main findings showed that both PE teachers and S&C coaches perceive a broad range of motor competencies important across all stages of development, but that the importance of motor competencies differ between stages of maturity and between participants. Additionally, qualitative findings highlighted several factors related to decision-making processes that explain PE teachers and S&C coaches’ perceptions of motor competency importance.

### Differences in PE teacher’s and S&C coaches’ perceptions between stages of maturity

The findings of the current study demonstrate that PE teachers and S&C coaches perceive a range of motor competencies to be important across all stages of maturity, consistent with previous research [27]. However, PE teachers and S&C coaches perceived importance of motor competencies differed across stages of maturity. Whilst some motor competencies (e.g., object control, balance, running, foundational movements for life) were perceived more important during childhood and pre-PHV, than circa- and post-PHV, the findings showed that the number of motor competencies identified as important by the majority (55–75%) and most (>75%) participants increased from childhood (i.e., PE = 10, S&C = 12) to post-PHV (PE = 15, S&C = 17). This finding contradicts previous theory suggesting that youths should develop a broad range of motor competencies during childhood, and transition towards sport specific competencies based on the training age/maturity status of the individual (e.g., Lloyd et al. [2], Barnett et al. [8], Gallahue and Ozmun [22]). Interestingly, qualitative responses representing PE teachers and S&C coaches understanding of themselves (e.g., existing values and beliefs) aligned with the theory, but not with their perceived importance, potentially highlighting differences in the importance ratings, knowledge, and application. For example, participants showed an understanding that motor competencies should progress towards sport specific skills in line with long-term athletic development models but apply this theory by perceiving more motor competencies are important as youths age and mature.

A second explanation for the limited agreement between importance and explanations for participants perceptions could be that contextual factors (e.g., policy, time, motivation), and how these are interpreted, also impact upon their perception of motor competence importance. For example, the national PE curriculum in the UK focuses upon teaching fundamental movement skills during key stage 1. During Key stage two, the focus becomes teaching these skills in isolation and in combination, along with developing flexibility, strength, technique, control, and balance through physical activities [29]. In key stages 3 and 4, the PE curriculum focuses on developing technical/tactical skills and performance through playing competitive sports, with less focus on motor competence [28]. This national education policy appears open for interpretation, and therefore, importance during childhood and pre-PHV stages may be dependent upon what PE teachers perceive as fundamental skills and/or what is currently practiced in their context. Recently, Hulteen et al. [5] questioned the “fundamental” approach, suggesting that it does not encapsulate the full range of skills required to promote physical activity participation across the lifespan. Instead, Hulteen et al. [5] proposed foundational movement skills, which combine traditional (e.g., locomotor, object control, stability skills) and less traditional (e.g., resistance training skills, cycling) motor competencies, to support and maximise opportunities for physical activity and sports participation. If UK policy adopted a foundational movement focus, PE teacher’s perceptions of importance of all movement competencies across all stages of development may be evident.

One key finding was that participants overlooked the importance of strength-based competencies (i.e., lower-body bilateral/unilateral, upper body pushing/pulling, core bracing) during early stages of maturity. Neither PE teachers nor S&C coaches rated these competencies as important during childhood, with PE teachers not rating these competencies as important until circa- and post-PHV. This finding supports previous evidence in soccer coaches showing that relatively few coaches support the introduction of strength training in younger populations, suggesting that current recommendations have little influence on their perceptions [45]. However, to effectively execute motor skills, a combination of cognitive processes, correct movement patterns, and force production and absorption are required [46, 47]. Therefore, developing competence in strength-based motor competencies during childhood and beyond should be viewed as important [47]. Kennedy et al. [48] have shown that an education programme directed at PE teachers in New Zealand can improve their knowledge, skills, techniques, and teaching strategies towards resistance training for youth. Therefore, educating UK-based PE teachers and S&C coaches on the benefits of strength-based motor competencies during childhood/pre-PHV may in turn shift their perceptions to reflect this. Consequently, if translated into practice, developing these competencies at earlier stages of maturity may lead to better movement mechanics, enhanced force production, reduced injury risk, and better health outcomes for youths [49].

### Comparing perceptions between PE teachers and S&C coaches

The findings of this study demonstrated that S&C coaches perceived more motor competencies as important than PE teachers across all stages of maturity. One explanation for this finding could be how policy dictates coaching practices, which subsequently influence perceptions. For example, the PE curriculum in the UK states that the aims of PE should be facilitated through physical activities and sports games (e.g., gymnastics, rounders, football) [28, 29], whereas S&C coaches are not bound by a curriculum, have more flexibility with how they coach, and may be more accustomed with constraints-based motor learning pedagogies (e.g., [47]). If PE teachers are bound to a games-based approach to teaching motor competencies through their curriculum, it may be challenging to develop a range of competencies (e.g., strength-based) that are not directly performed within sports games. Thus, PE teachers may perceive fewer competencies as important. This point is substantiated by the second order theme whereby participants perceptions were influenced by what they coach. If PE teacher’s perceptions are replicated within coaching practices, youths who are exposed to PE alone may only develop a limited range of motor competencies, which could lead to reduced levels of actual and perceived motor competence, physical fitness, and physical activity engagement as youths develop [14, 15]. Therefore, this evidence suggests a need for updated methods to facilitate the development of a broad range of motor competencies in youths, which could be facilitated through teacher-coach collaboration (e.g., integration of S&C principles into the PE curriculum through enhanced professional training/education for teachers [16]).

Although neither participant group rated strength-based competencies as important during childhood, S&C coaches were close to a majority agreement for the importance of these competencies during childhood (agreement = 42–54% scoring four or five) and reached a majority agreement of their importance during pre-PHV (agreement = 56–77% scoring four or five). In comparison, PE teachers rated strength-based motor competencies significantly less important than S&C coaches during childhood and pre-PHV. One explanation for the differences in PE teachers and S&C coaches’ perceptions during childhood and pre-PHV may be due to the myths and misconceptions of strength training for youths indicated by PE teachers in this study. Similar misconceptions are apparent within soccer coaches [45] and are an apparent barrier to implementing strength-based motor competencies in schools, along with staffing issues, time, and motivation [48]. This barrier is hampered further within primary education, with teachers being ill prepared to teach physical education [50], where inadequate standards of PE provision in primary schools is attributed to limited subject knowledge [51]. This further strengthens the need for teacher/coach education programmes that disprove myths and misconceptions, and develop knowledge about the benefits of developing all motor competencies within youth.

Both PE teachers and S&C coaches had high agreement for the importance of fundamental movements skills (running, jumping, landing, balance, gymnastics and other locomotion, object control) and foundational movements for life at early stages of maturity (PE teachers = 56–87%; S&C coaches = 73–94% scoring four or five across childhood and pre-PHV). The similarities between PE teachers and S&C coaches scores for these competencies during childhood and pre-PHV could be explained by how these individuals coach. Both PE teachers and S&C coaches referred to utilising fun games (i.e., their pedagogy) within younger cohorts to allow movement exploration within their individuals. This could be reflective of an oversimplified understanding and application of the FUNdamental stage within the popularised long-term *athlete* development model [52], which emphasises developing fundamental movement skills through fun and engaging activities. Indeed, this approach is deemed good practice, as exploratory activities stimulate creativity, enhance enjoyment, and increase motivation within children [26]. The Balyi and Hamilton [52] model was previously adopted within national governing body coaching qualifications [e.g., 53–55], and was an important predecessor to more recent evidence-based models [e.g., the Youth Physical Development model; 30]. Newer models of long-term athletic development suggest that motor competence, strength, and other physical/psycho-social qualities should be developed simultaneously [30]. Thus, utilising methods that combine the development of these qualities [e.g., AMSC, 26, 46] should be a priority, and can be facilitated through competency-driven and maturity-appropriate prescription [e.g., 47]. However, further research is required to highlight PE teachers and S&C coaches understanding of long-term athletic development models, and how they influence perceptions of motor competency importance.

By post-PHV, PE teachers rated balance, foundational movements for life, gymnastics and other locomotion, and object control more important than S&C coaches. Within the qualitative responses, the utilisation of past experiences stands out as potential reasons for this difference. For example, one PE teacher discussed how the motor competence of adolescents has worsened over their time as a teacher, leading to basic competencies being perceived as important during adolescence. The responses from this teacher provides anecdotal support towards data indicating that adolescent rugby union players [56] and cricketers [57] lack proficiency in some motor competencies (e.g., sprinting, squatting), and that secular trends are showing that youths motor competence and physical fitness are declining [58–60]. With participants drawing on past experiences it is evident that, for some, perceptions are not just formed on the premise of knowledge, understanding of theory, and education, but also through continual reflection and learning. Therefore, to aid reflection and learning, PE teachers and S&C coaches should longitudinally track motor competence from a process (technique) and product (outcome) perspective. Process and product evaluations provide detailed insights into motor competence as youth develop [61, 62], and could highlight the individual needs of children and adolescents [1]. Moreover, adopting a session planning framework that aids session-by-session reflection may also be of use. For example, the RAMPAGE (raise, activate, mobilise, prepare, activity, games, evaluate) coaching session framework facilitates effective planning, delivery, and evaluation of long-term athletic development sessions [63]. Here, the RAMP section provides coaches with the ability to integrate motor competencies into a structured warm up, which may provide the opportunity to target motor competencies that PE teachers and S&C coaches deem less important (e.g., fundamental/foundational competencies during post-PHV) on a more regular basis [27]. The activity and games sections of the framework then provide a basis to target more important motor competencies, while the evaluation element allows individuals to reflect and learn from their delivery [63], and continually shape their perceptions based on their coaching experience.

### Limitations

Whilst this study is the first to explore PE teachers and S&C coaches’ perspectives of motor competencies across stages of maturity, it is not without limitations. Firstly, the findings from this study only represent PE teachers and S&C coaches based in the UK and cannot be generalised to individuals from other countries. Second, an initial exploratory view of qualitative responses did not provide a full understanding of PE teachers’ and S&C coaches’ perceptions, because some qualitative explanations appeared contradictory to quantitative perceptions. As such, more work is required to understand PE teacher’s and S&C coaches’ perceptions further, which differentiates between PE teachers and S&C coaches and examines the contradictions between quantitative and qualitative data. Thirdly, due to the volume of data presented, it was outside the scope of this study to conduct further analyses to explore differences in perceptions between types of school (e.g., primary vs. secondary; state vs. independent school) or by S&C coach environment (e.g., team sport vs. individual sport). However, this novel study is the first to provide detailed analysis of differences in perceptions between stages of maturity, and between PE teachers and S&C coaches. Further, this study expands on the limitations of previous research in S&C coaches by using a mixed-method approach. Future research may seek to evaluate the perceptions of other stakeholders involved in long-term athletic development (e.g., sports coaches, parents), and compare perceptions of important motor competencies between teachers and coaches from a variety of countries/environments/settings. Additionally, the use of more in-depth qualitative methods (e.g., interviews/focus groups) may be key to further understand the perceptions and practices for developing motor competencies in more detail and may inform how these perceptions have developed over time.

## Conclusion

The aims of this study were to evaluate PE teachers and S&C coaches’ perceptions of important motor competencies, compare perceptions by maturity status and between participants, and explore factors that influence PE teachers’ and S&C coaches’ perceptions of motor competencies. Findings showed that both PE teachers and S&C coaches perceived a broad range of motor competencies important across all stages of development, with S&C coaches perceiving more motor competencies important than PE teachers across all stages of maturity. This finding indicates that youth who are exposed to PE alone may only develop a limited range of motor competencies, and that multiple individuals and settings may be required to adequately facilitate motor competence development for youths (e.g., S&C coach/PE teacher collaboration to integrate S&C principles into the PE curriculum; extra-curricular athletic development lessons; athletic development in sports clubs). Further findings indicate that neither PE teachers nor S&C coaches rated strength-based competencies important during childhood, with PE teachers rating such competencies less important than S&C coaches until circa-PHV. Qualitative findings indicated that some PE teachers perceived there to be risks associated with developing strength-based competencies during early stages of maturity, and that PE teachers and S&C coaches may need education on the importance of developing strength-based motor competencies early in youth development.

Physical Education teachers and S&C coaches similarly rate fundamental and foundational competencies important during childhood, likely due to the use of fun games to facilitate motor competence development in this cohort. Nevertheless, it may be of benefit to convert evidence-based approaches [e.g., AMSC; 47] into translatable resources to assist PE teachers and S&C coaches with implementing more strength-based competencies into exploratory activities during childhood and pre-PHV. Mixed-model analysis highlighted that PE teachers perceive fundamental and foundational competencies more important than S&C coaches during post-PHV. This result may be due to a combination of S&C coaches expecting to develop more advanced competencies at this stage, and PE teachers articulating their perceptions based on past experiences. This finding suggests that PE teachers and S&C coaches’ perceptions may not be reflective of knowledge and education alone, but also through continual reflection and learning. Regularly assessing youths motor competencies and implementing short-, medium- and long-term planning and reflections may provide PE teachers and S&C coaches more opportunities to shape their perceptions of important motor competencies for all youths, hence improving the competencies delivered within youths across all stages of maturity. PE teachers, S&C coaches, researchers, and other stakeholder may utilise the list of motor competencies presented to reflect on their practices, to design motor competence interventions, or for future research design and methodology in this space.
